# The m^6^A Methyltransferase METTL3 Ameliorates Methylglyoxal-Induced Impairment of Insulin Secretion in Pancreatic β Cells by Regulating MafA Expression

**DOI:** 10.3389/fendo.2022.910868

**Published:** 2022-07-08

**Authors:** Yi Cheng, Xin-Ming Yao, Si-Min Zhou, Yue Sun, Xiang-Jian Meng, Yong Wang, Yu-Jie Xing, Shu-Jun Wan, Qiang Hua

**Affiliations:** ^1^ Department of Endocrinology, The First Affiliated Hospital of Wannan Medical College, Yijishan Hospital, Wuhu, China; ^2^ Key Laboratory of Non-coding RNA Transformation Research of Anhui Higher Education Institution, Wannan Medical College, Wuhu, China; ^3^ Central Laboratory of Yijishan Hospital, The First Affiliated Hospital of Wannan Medical College, Wuhu, China; ^4^ Clinical Research Center for Critical Respiratory Medicine of Anhui Province, Wannan Medical College, Wuhu, China

**Keywords:** METTL3, methylglyoxal, N^6^-methyladenosine, pancreatic β cells, insulin secretion

## Abstract

Methylglyoxal, a major precursor of advanced glycation end products, is elevated in the plasma of patients with type 2 diabetes mellitus. Islet β-cell function was recently shown to be regulated by N^6^-methyladenosine (m^6^A), an RNA modification consisting of methylation at the N6 position of adenosine. However, the role of m^6^A methylation modification in methylglyoxal-induced impairment of insulin secretion in pancreatic β cells has not been clarified. In this study, we showed that treatment of two β-cell lines, NIT-1 and β-TC-6, with methylglyoxal reduced m^6^A RNA content and methyltransferase-like 3 (METTL3) expression levels. We also showed that silencing of METTL3 inhibited glucose-stimulated insulin secretion (GSIS) from NIT-1 cells, whereas upregulation of METTL3 significantly reversed the methylglyoxal-induced decrease in GSIS. The methylglyoxal-induced decreases in m^6^A RNA levels and METTL3 expression were not altered by knockdown of the receptor for the advanced glycation end product but were further decreased by silencing of glyoxalase 1. Mechanistic investigations revealed that silencing of METTL3 reduced m^6^A levels, mRNA stability, and the mRNA and protein expression levels of musculoaponeurotic fibrosarcoma oncogene family A (MafA). Overexpression of MafA greatly improved the decrease in GSIS induced by METTL3 silencing; silencing of MafA blocked the reversal of the MG-induced decrease in GSIS caused by METTL3 overexpression. The current study demonstrated that METTL3 ameliorates MG-induced impairment of insulin secretion in pancreatic β cells by regulating MafA.

## Introduction

Type 2 diabetes mellitus (T2DM) is a public health problem of considerable magnitude that is characterized by hyperglycemia, insulin resistance, and gradual exhaustion of insulin secretion from pancreatic β cells (1). Methylglyoxal (MG), a highly reactive dicarbonyl product of glucose metabolism, is believed to be the most important precursor of advanced glycation end products (AGEs). We (2) and others (3, 4) have demonstrated that plasma MG levels are significantly higher in T2DM patients than in control individuals. MG may be involved in the development of DM and diabetic complications by acting as either a precursor of AGEs or a direct toxin (5–7). MG reduced islet β-cell insulin secretion both *in vivo* and *in vitro* (8–10); however, the molecular mechanism by which MG treatment results in decreased insulin secretion has not been elucidated.

N^6^-Methyladenosine (m^6^A), the most frequent mRNA modification in eukaryotes, has garnered wide interest in recent years because of its roles in regulating mRNA splicing, output, translation, and stability (11, 12). m^6^A levels are mainly regulated by methyltransferases such as methyltransferase-like 3 (METTL3) and methyltransferase-like 14 (METTL14), as well as demethylases, fat mass, obesity-associated protein (FTO), and α-ketoglutarate-dependent dioxygenase homolog 5 (ALKBH5) (13).

The m^6^A modification is essential for the physiological function of pancreatic β cells (14). Levels of m^6^A RNA in the pancreatic islets and plasma of patients with T2DM were markedly lower than those in control subjects (14–18). These changes in m^6^A levels were attributed to decreases in METTL3 and METTL14 expression (14, 18–20) and an increase in FTO expression (16). Musculoaponeurotic fibrosarcoma oncogene family A (MafA), a key regulator of insulin gene transcription, is markedly decreased in the β cells of patients with T2DM (21). Wang et al. showed that METTL3 specifically targets MafA and regulates its protein expression (18). However, to the best of our knowledge, it remains unclear whether the m^6^A modification is involved in MG-induced dysfunction of β-cell insulin secretion. Therefore, the present study was designed to explore the connection between MG and m^6^A levels and to clarify the mechanisms underlying the role of the m^6^A RNA modification in MG-induced β-cell dysfunction.

## Materials and Methods

### Cell Culture

The mouse insulinoma β-cell lines NIT-1 and β-TC-6 were purchased from Procell Life Science and Technology Co. (Wuhan, China). The cells were cultured in Dulbecco’s modified Eagle’s medium (DMEM; Gibco, California, USA) containing 10% fetal bovine serum (Gibco, California, USA). NIT-1 and β-TC-6 cells were treated with 1 mM of MG (Sigma, Missouri, USA) for the duration indicated in each experiment. This concentration of MG was selected based on previous studies (9, 10), which showed that 1 mM of MG sufficiently decreased islet β-cell function *in vitro.*

### Cell Transfection

Small interfering RNAs (siRNAs) targeting METTL3 (si-METTL3), the receptor for advanced glycation end products (RAGE, si-RAGE), glyoxalase 1 (Glo-1, si-Glo-1), and MafA (si-MafA), as well as a negative control siRNA (si-NC), were synthesized by Riobio Technology Co. (Guangzhou, China). The siRNA sequences were as follows:

siRNA METTL3-1: 5′‐GGACTCGACTACAGTAGCT‐3′;
siRNA METTL3-2: 5′‐CAAGTATGTTCACTATGAA‐3′;
siRNA METTL3-3: 5′‐GACTGCTCTTTCCTTAATA‐3′;
siRNA RAGE-1: 5′‐GCATTCAGCTGTTGGTTGA‐3′;
siRNA RAGE-2: 5′‐CCACTGGAATTGTCGATGA‐3′;
siRNA RAGE-3: 5′‐CCAGCAGCTAGAATGGAAA‐3′;
siRNA Glo-1: 5′‐CTATGAAGTTCTCGCTCTA‐3′;
siRNA Glo-2: 5′‐GCAAACGATGCTAAGAATT‐3′;
siRNA Glo-3: 5′‐AGAAGACAGCATGGACGTT‐3′;
siRNA MafA-1: 5′‐TCAACGACTTCGACCTGAT‐3′;
siRNA MafA-2: 5′‐TGATGAAGTTCGAGGTGAA‐3′;
siRNA MafA-3: 5′‐GATGAAGTTCGAGGTGAAG‐3′.

Lipofectamine 3000 reagent (Invitrogen, California, USA), Opti-MEM medium (Gibco, California, USA), and siRNAs were mixed and incubated at room temperature for 15 min and then added to cells and incubated for 36 h. Three siRNA sequences were synthesized for each target gene, and the siRNA targeting METTL3-2, RAGE-1, Glo-1-2, and MafA-3 with the highest inhibition efficiencies were selected for subsequent experiments (**Supplementary Figure S1**).

Recombinant adenovirus constructs with either METTL3 (Ad-METTL3) or an empty vector (Ad-NC) and pCDNA3.1 plasmids carrying either MafA (pCDNA-MafA) or the empty vector (pCDNA) were synthesized by HanBio Technology Co. (Shanghai, China). Cells were infected with Ad-NC or Ad-METTL3 for 48 h. Cells were transfected with pCDNA or pCDNA-MafA using Lipofectamine 3000 reagent (Invitrogen, California, USA) for 48 h.

### m^6^A RNA Methylation Quantification

m^6^A RNA methylation was quantified using the m^6^A RNA Methylation Quantification Kit (Abcam, Cambridge, UK). Total RNA was extracted from NIT-1 and β-TC-6 cells using TRIzol reagent (Tiangen, Beijing, China). Briefly, the negative control, positive control, and 200 ng of sample RNA were added to the designated wells. Diluted capture antibody, detection antibody, and diluted enhancer solution were then added to each well. The m^6^A content was quantified colorimetrically; the absorbance at 450  nm was measured using a microplate reader; and the m^6^A content was calculated based on a standard curve. The percentage of total RNA containing m^6^A was calculated using the formula provided by the manufacturer.

### Quantitative Real-Time PCR

Total RNA was extracted and reverse transcribed using a reverse transcription kit (Tiangen, Beijing, China) according to the manufacturer’s instructions. Gene expression was analyzed by qPCR using the SYBR Green PCR Kit (Tiangen, Beijing, China). Expression levels of target genes were normalized to β-actin and differences were calculated using the 2^−ΔΔCt^ method. The primer sequences used were as follows:

METTL3 forward primer: 5′-CATCCGTCTTGCCATCTCTACGC-3′,

reverse primer: 5′-GCAGACAGCTTGGAGTGGTCAG-3′;

METTL14 forward primer: 5′-TCGACCGAAGTCACCTCCTC-3′,

reverse primer: 5′-AGGAGTAAAGCCGCCTCTGT-3′;

FTO forward primer: 5′-GACACTTGGCTTCCTTACCTGACC-3′,

reverse primer: 5′-ACCTCCTTATGCAGCTCCTCTGG-3′;

ALKBH5 forward primer: 5-′GCAAGGTGAAGAGCGGCATCC-3′,

reverse primer: 5′-GTCCACCGTGTGCTCGTTGTAC-3′;

MafA forward primer: 5′-GCTTCAGCAAGGAGGAGGTCAT-3′,

reverse primer: 5′-TCTCGCTCTCCAGAATGTGCCG-3′;

β-Actin forward primer: 5′-CGTGAAAAGATGACCCAGATCA-3′,

reverse primer: 5′-CACAGCCTGGATGGCTACGT-3′.

### Western Blot Analysis

NIT-1 and β-TC-6 cells were washed twice with cold PBS and lysed in 100 μl of modified RIPA buffer (Beyotime, Shanghai, China). Proteins were separated using SDS-PAGE and immediately transferred to nitrocellulose membranes. The membranes were incubated with the primary antibody overnight and then with the appropriate secondary antibodies for 2 h. The antibodies used were as follows: anti-METTL3 (Cat. No.: ab195352, 1:1000; Abcam, Cambridge, UK), anti-METTL14 (Cat. No.: ab220030, 1:1000; Abcam, Cambridge, UK), anti-RAGE (Cat. No.: 42544, 1:800; CST, Massachusetts, USA), anti-Glo-1 (Cat. No.: NP-006699.2, 1:500; ABclonal, Wuhan, China), anti-MafA (Cat. No.: 79737, 1:1000; CST, Massachusetts, USA), and anti-β-actin (Cat. No.: T0022, 1:3000; Affinity, Melbourne, Australia). The target proteins on the blots were detected using a Tanon 5200 visualizer. The results were assessed by densitometry using ImageJ software.

### Glucose-Stimulated Insulin Secretion

As described in our previous study, cultured cells were washed with Krebs buffer (128.8 mM of NaCl, 4.8 mM of KCl, 1.2 mM of MgSO_4_, 1.2 mM of KH_2_PO_4_, 1.2 mM of CaCl_2_, and 10 mM of HEPES, pH 7.4) containing 0.2% bovine serum albumin. NIT-1 cells were incubated in Krebs buffer containing 2.8 mM of glucose for 30 min, and basal insulin secretion was measured (22–24). Stimulated insulin secretion was measured after incubating NIT-1 cells in Krebs buffer with 25 mM of glucose for 60 min. An aliquot of the buffer was collected, and insulin release was measured using an ELISA kit (Abcam, Cambridge, UK). The glucose-stimulated insulin secretion (GSIS) index was calculated by dividing the insulin secreted in cells exposed to 25 mM of glucose by the insulin secreted in cells exposed to 2.8 mM of glucose (24).

### Methylated RNA Immunoprecipitation Coupled With qPCR Assay

Methylated RNA immunoprecipitation coupled with qPCR (MeRIP-qPCR) was performed using the MeRIP kit (Bersinbio, Guangzhou, China), according to the manufacturer’s instructions. Briefly, total RNA was extracted from NIT-1 cells using TRIzol reagent, and the extracted RNA was fragmented using ultrasound treatment. The processed fragments were approximately 300 bp. After fragmentation, 50 μl of each RNA sample (the input sample) was stored at −80°C and the remaining portion of each RNA sample was immunoprecipitated with an anti-m^6^A antibody (Abcam, Cambridge, UK) or anti-IgG antibody. The RNA-antibody hybridization solution was incubated with Protein A/G magnetic beads for 1 h at 4°C in a vertical mixer. The beads were washed three times and digested with proteinase K at 55°C for 45 min. The supernatant was transferred to new RNase-free tubes, and the RNA was purified and subjected to qPCR. The MafA primer sequences were as follows:

Forward: 5′-CAGGAAAAGCGGTGCTGGAGG-3′,

Reverse: 5′-CGAAGCTCTGACCCCGGAAGG-3′.

### RNA Stability Assay

NIT-1 cells were treated with 5 μg/ml actinomycin D (Sigma, Missouri, USA) to inhibit mRNA transcription. After incubation for the indicated times, the treated cells were collected, and total RNA was extracted using TRIzol reagent. MafA mRNA expression was measured by qPCR. β-Actin was used for normalization.

### Statistical Analysis

Data are presented as the mean ± standard deviation (SD). One-way analysis of variance followed by the Newman–Keuls test was used to compare differences among groups. Statistical significance was set at *p* < 0.05.

## Results

### m^6^A RNA Modification and METTL3 Expression Levels Were Reduced in MG-Treated Pancreatic β Cells

**Figure 1:**
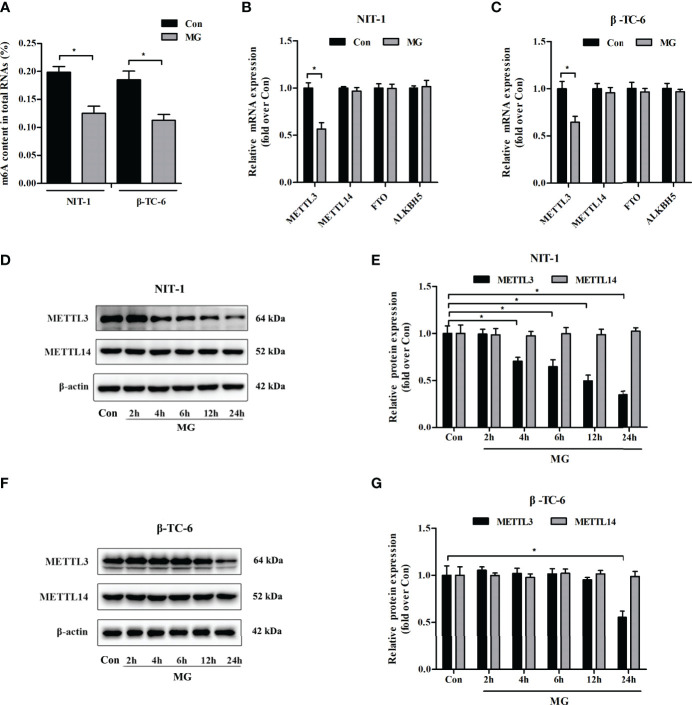
Methylglyoxal (MG) treatment decreased m^6^A RNA methylation and METTL3 expression levels in pancreatic β cells. **(A)** m^6^A levels in total RNA from NIT-1 and β-TC-6 pancreatic β cells treated with 1 mM of MG (MG) for 24 h and untreated control cells (Con). **(B, C)** mRNA expression of the m^6^A methyltransferases METTL3 and METTL14 and the demethylases FTO and ALKBH5 in NIT-1 **(B)** and β-TC-6 **(C)** cells treated with 1 mM of MG for 24 h as measured by qPCR. The mRNA level of each gene was normalized to β-actin. **(D–G)** Immunoblotting of METTL3 and METTL14 protein expression levels in NIT-1 **(D, E)** and β-TC-6 **(F, G)** cells treated with 1 mM of MG for different time periods. β-Actin was used as an internal control. Results are presented as the means ± SD of 3–4 independent experiments. ^*^ *p* < 0.05.

To explore the potential role of MG in the m^6^A modification in pancreatic β cells, the m^6^A content in total RNA was measured in MG-treated and untreated NIT-1 and β-TC-6 cells. As shown in **Figure 1A**, the m^6^A levels in RNA were significantly reduced in NIT-1 (reduced by 36.8%) and β-TC-6 (reduced by 39.3%) cells after MG treatment, indicating an MG-induced decrease in m^6^A modification in pancreatic β cells. We then evaluated the mRNA and protein expression of the m^6^A methyltransferases METTL3 and METTL14 and the demethylases FTO and ALKBH5 in MG-treated and untreated NIT-1 and β-TC-6 cells. Following treatment with MG for 24 h, METTL3 mRNA levels were markedly downregulated in both NIT-1 cells (reduced by 43.5% versus the untreated control (Con), *p* < 0.05) and β-TC-6 cells (reduced by 35.5% versus Con, *p* < 0.05) (**Figures 1B, C** **)**. In contrast, the mRNA expression levels of METTL14, FTO, and ALKBH5 did not change significantly (**Figures 1B, C** **)**. Immunoblotting analysis showed that MG treatment decreased METTL3 protein expression in NIT-1 cells in a time-dependent manner (reduced by 29.4% at 4 h, 35.3% at 6 h, 50.4% at 12 h, and 65.2% at 24 h versus Con, *p* < 0.05; **Figure 1D**). In contrast, in MG-treated β-TC-6 cells, METTL3 protein expression was only reduced after 24 h of treatment (reduced by 44.5% versus Con, *p* < 0.05; **Figure 1F**). These findings suggest that MG may reduce m^6^A levels in pancreatic β cells by decreasing METTL3 expression; NIT-1 cells are more sensitive than β-TC-6 cells to this MG treatment-induced decrease in METTL3 expression. Therefore, we selected NIT-1 cells to further characterize this effect.

### Effects of METTL3 on Glucose-Stimulated Insulin Secretion From Pancreatic β Cells

**Figure 2:**
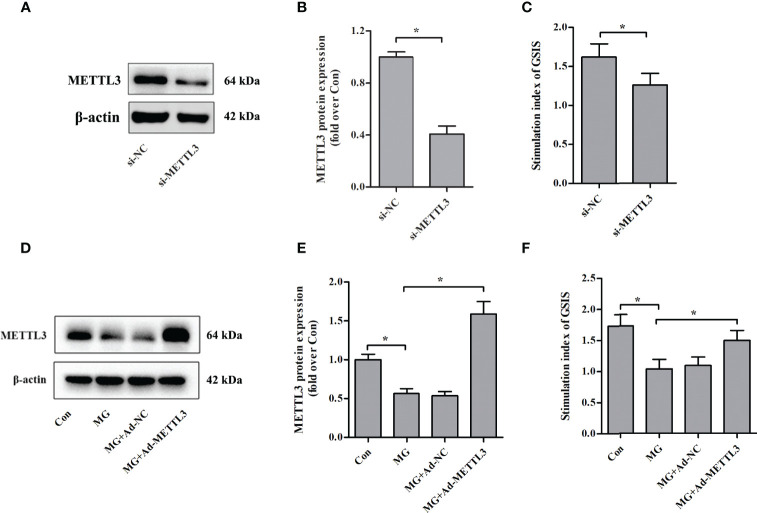
Effects of METTL3 on GSIS from pancreatic β cells. **(A, B)** Immunoblot of METTL3 protein expression in NIT-1 cells transfected with METTL3 siRNA (si-METTL3) or a nonspecific control siRNA (si-NC), which was set to 1. **(C)** The GSIS index of NIT-1 cells transfected with si-METTL3 or si-NC. **(D, E)** Immunoblot of METTL3 protein expression in NIT-1 cells (MG), NIT-1 cells transfected with METTL3 expression adenovirus (MG+Ad-METTL3), and NIT-1 cells transfected with a nonspecific control adenovirus (MG+Ad-NC) that were treated with 1 mM of MG for 24 h. **(F)** The GSIS index in NIT-1 cells was transfected with Ad-METTL3 or Ad-NC and treated with 1 mM of MG for 24 h. Results are presented as the means ± SD of *n* = 3–4 independent experiments. ^*^ *p* < 0.05.

To clarify the role of METTL3 in β-cell insulin secretion, we assessed the effects of METTL3 on GSIS from NIT-1 cells under normal culture conditions. We suppressed the expression of METTL3 with siRNA (**Figures 2A, B** **)** and found that the GSIS index was significantly reduced (by 22.2%, *p* < 0.05 versus si-NC, **Figure 2C**). To further investigate the biological function of METTL3, an adenovirus vector to overexpress METTL3 was transfected into NIT-1 cells (**Figures 2D, E** **)**. Upregulation of METTL3 significantly reversed the MG-induced reduction in the GSIS index in NIT-1 cells (by 44.2%, *p* < 0.05; **Figure 2F**).

### Effects of RAGE Knockdown on METTL3 Expression and m^6^A RNA Levels in NIT-1 Cells

**Figure 3:**
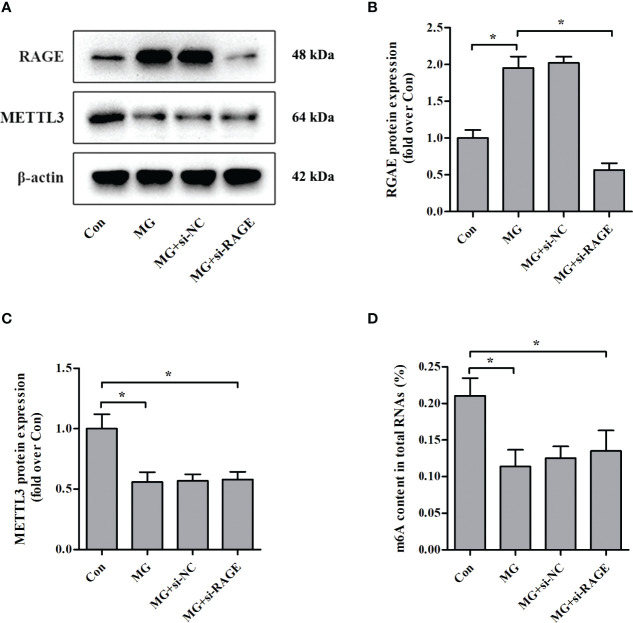
Effects of RAGE knockdown on METTL3 expression and m^6^A RNA methylation levels in NIT-1 cells. **(A–C)** Immunoblotting of RAGE and METTL3 protein expression in NIT-1 cells transfected with RAGE siRNA (si-RAGE) or a nonspecific control siRNA (si-NC) and treated with 1 mM of MG for 24 h. **(D)** m^6^A levels in total RNA from NIT-1 cells treated as described for **(A–C)**. Results are presented as the means ± SD of *n* = 3–4 independent experiments. ^*^ *p* < 0.05.

Advanced glycation end products (AGEs) exert biological effects *via* specific receptors; the most well-characterized is RAGE (25). Treatment of NIT-1 cells with MG, a major precursor of AGEs, enhanced RAGE expression (**Figures 3A, B** **)**. To investigate whether the effects of MG on the m^6^A modification are mediated by RAGE, we knocked down RAGE expression using a siRNA (**Figures 3A, B** **)**. The MG-induced reductions in METTL3 expression (**Figures 3A, C** **)** and m^6^A RNA modification levels (**Figure 3D**) were not reversed by knockdown of RAGE.

### Effects of Glo-1 Knockdown on METTL3 Expression and m^6^A Levels in NIT-1 Cells

**Figure 4:**
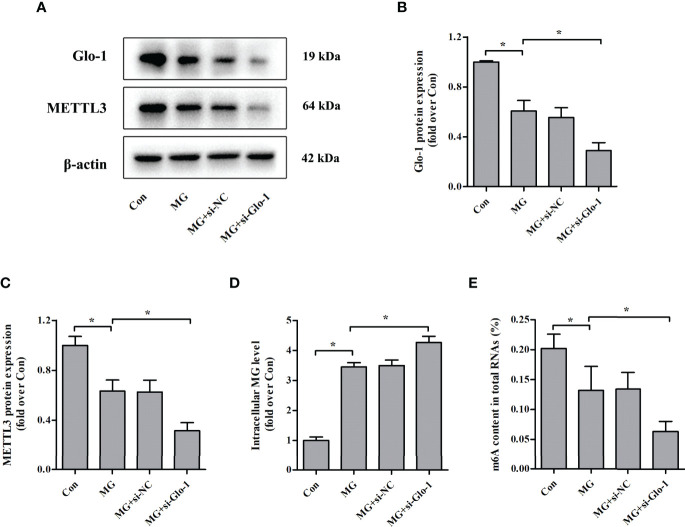
Effects of Glo-1 knockdown on METTL3 expression and m^6^A RNA methylation levels in NIT-1 cells. **(A–C)** Immunoblotting of Glo-1 and METTL3 protein expression in NIT-1 cells transfected with Glo-1 (si-Glo-1) or a nonspecific control siRNA (si-NC) and treated with 1 mM of MG for 24 h. **(D)** Intracellular MG levels in NIT-1 cells were treated as described in **(A–C)**. **(E)** m^6^A levels in total RNA from NIT-1 cells treated as described in **(A–C)**. Results are presented as the means ± SD of *n* = 3–4 independent experiments. ^*^ *p* < 0.05.

Glo-1 is the main component of the glyoxalase system and is essential for MG detoxification in all mammalian cells (7). Similar to previous studies (26–29), MG treatment decreased Glo-1 expression in NIT-1 cells (**Figures 4A, B** **)**. Interestingly, Glo-1 knockdown further reduced METTL3 expression (decreased by 50.2% versus MG treated, *p* < 0.05; **Figures 4A, C** **)** and m^6^A RNA levels (decreased by 52.3% versus MG treated, *p* < 0.05; **Figure 4E**) in MG-treated NIT-1 cells. These effects were attributed to decreased MG degradation and an increased intracellular MG concentration (increased by 23.7% versus MG treated, *p* < 0.05; **Figure 4D**).

### Loss of METTL3 Attenuated the Expression of MafA

**Figure 5:**
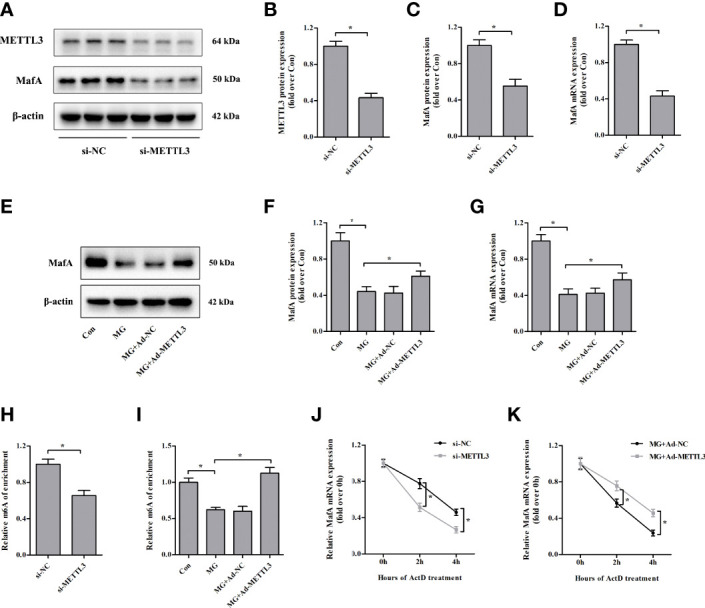
Loss of METTL3 attenuates the expression of MafA. **(A–D)** METTL3 and MafA protein and mRNA levels were measured by immunoblotting and qPCR, respectively, in NIT-1 cells transfected with METTL3 siRNA (si-METTL3) or a nonspecific control siRNA (si-NC), which was set to 1. **(E–G)** MafA protein and mRNA expression levels in NIT-1 cells transfected with Ad-METTL3 or a nonspecific control adenovirus (Ad-NC) and treated with 1 mM of MG for 24 h were measured by immunoblotting and qPCR, respectively. **(H, I)** m^6^A MafA mRNA levels as detected by MeRIP-qPCR in NIT-1 cells transfected with si-METTL3 or si-NC, which was set to 1 **(H)**, or Ad-METTL3 or Ad-NC and treated with 1 mM of MG for 24 h **(I)**. **(J, K)** MafA mRNA as measured by qPCR in NIT-1 cells transfected with si-METTL3, si-NC, Ad-METTL3, or Ad-NC and treated with ActD to block transcription. Results are presented as the means ± SD of *n* = 3–4 independent experiments. ^*^ *p* < 0.05.

Similar to the results observed in specific β cells in the islets of METTL3/14 knockout mice (18), we found that MafA protein and mRNA expression levels were markedly downregulated in NIT-1 cells after METTL3 knockdown (decreased by 44.6% and 57.0%, respectively, versus si-NC, *p* < 0.05; **Figures 5A–D**). We conducted rescue experiments and observed that overexpression of METTL3 reversed the decreases in MafA protein and mRNA expression in MG-treated NIT-1 cells (increased by 38.5% and 39.1%, respectively, versus MG, *p* < 0.05; **Figures 5E-G**). MeRIP-qPCR confirmed that the m^6^A levels in MafA mRNA were decreased by METTL3 silencing in NIT-1 cells under normal culture conditions (decreased by 34.6% versus si-NC, *p* < 0.05; **Figure 5H**). The m^6^A levels in MafA mRNA were increased by upregulation of METTL3 in MG-treated NIT-1 cells (increased by 81.2% versus MG, *p* < 0.05; **Figure 5I**). We conducted an RNA stability assay to investigate the relationship between m^6^A and MafA mRNA stability. As shown in **Figure 5J**, MafA mRNA levels were decreased in METTL3-silenced NIT-1 cells after ActD treatment, indicating METTL3 knockdown led to reduced stability of MafA mRNA. MafA mRNA decay induced by MG treatment in NIT-1 cells was significantly ameliorated by transfection with Ad-METTL3 (**Figure 5K**). These results indicate that METTL3 regulates MafA expression in an m^6^A-dependent manner.

### A Change in MafA Expression Was Associated With METTL3-Regulated GSIS From NIT-1 Cells

**Figure 6:**
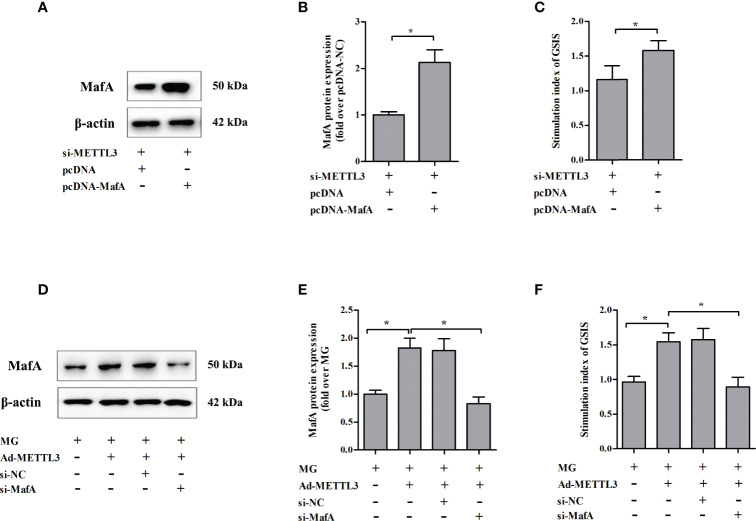
Changes in MafA expression were associated with METTL3-regulated GSIS from NIT-1 cells. **(A)** Immunoblot of MafA expression in NIT-1 cells transfected with si-METTL3 + pcDNA or si-METTL3 + pcDNA MafA. **(B)** Quantification of the immunoblot in **(A)**. **(C)** The GSIS index of the cells described in **(A)**. **(D)** Immunoblot of MafA expression in NIT-1 cells transfected with Ad-METTL3 + si-NC or Ad-METTL3 + si-MafA and treated with 1 mM MG for 24 h. **(E)** Quantification of the immunoblot in **(D)**. **(F)** The GSIS index of the cells described in **(D)**. Results are presented as means ± SD of *n* = 3‐4 independent experiments. ^*^ *p* < 0.05.

To test whether METTL3 regulates GSIS from NIT-1 cells through MafA, rescue experiments were conducted by transfecting NIT-1 cells transfected with both si-METTL3 and either pcDNA or pcDNA MafA (**Figures 6A–C**). Overexpression of MafA (pcDNA MafA) greatly improved the decrease in GSIS triggered by METTL3 silencing in NIT-1 cells (increased by 36.7% versus si-METTL3 + pcDNA, *p* < 0.05). NIT-1 cells were transfected with Ad-METTL3 + si-NC or Ad-METTL3 + si-MafA and the GSIS index was measured (**Figures 6D–F**). The reversal of the MG-induced GSIS decrease caused by METTL3 overexpression in NIT-1 cells was abrogated by knockdown of MafA (decreased by 42.4% compared to MG + Ad-METTL3, *p* < 0.05).

## Discussion

Accumulation of MG in plasma has been implicated in the development of both DM and diabetic complications (5–7). We previously showed that plasma MG levels are markedly enhanced in patients with newly diagnosed T2DM, indicating that MG accumulation plays an important role in the onset of DM and not merely its complications (2). In fact, MG levels are increased and insulin content and GSIS were reduced in pancreatic islets isolated from a rat model with chronic MG infusion-induced T2DM, suggesting that MG accumulation leads to pancreatic β-cell dysfunction in T2DM (8). Therefore, the current study was designed to explore the regulatory mechanisms of MG β-cell dysfunction.

Although increasing evidence suggests that m^6^A plays a role in many pathological processes in eukaryotic cells, studies on its roles in controlling pancreatic β-cell maturity and physiological function have just begun (14). We reported here, for the first time, that MG treatment significantly decreased m^6^A levels in NIT-1 and β-TC-6 cells. Although MG treatment had no effect on the expression of METTL14, FTO, and ALKBH5, it obviously reduced METTL3 mRNA and protein expression. METTL3 and METTL14 form stable heterodimers and maintain high levels of m^6^A (30). METTL3 may be more important for regulating pancreatic β-cell function than METTL14 because the increase in blood glucose is higher in β-cell METTL3 knockout mice than in β-cell METTL14 knockout mice (19, 20). Therefore, the decrease in m^6^A levels in β cells was attributed to MG-induced downregulation of METTL3 expression. Silencing of METTL3 impaired GSIS from NIT-1 cells under normal culture conditions, whereas upregulation of METTL3 in NIT-1 cells ameliorated the MG-induced decrease in GSIS. These data suggest that METTL3 plays a significant role in MG-induced reductions in pancreatic β-cell m^6^A levels and GSIS.

AGE-RAGE interaction stimulates the generation of reactive oxygen species and inflammation mechanisms that enhance AGE-induced cell and tissue injury (25). MG can increase AGE accumulation and RAGE expression, resulting in human endothelial cell injury (31, 32). RAGE-deficient mice have characteristics that antagonize the decrease in insulin sensitivity caused by MG administration (33). Thus, we investigated whether the MG-induced changes in m^6^A levels and METTL3 expression were associated with RAGE. However, RAGE silencing failed to reverse the MG-induced reductions in METTL3 expression and m^6^A levels in RNA, indicating that these m^6^A changes are not closely related to the RAGE pathway.

As described in our previous studies (34) and others (9), incubation of pancreatic β cells with MG dramatically increased intracellular MG content. Further experiments were performed to determine whether the intracellular accumulation of MG in NIT-1 cells is involved in the m^6^A-related changes caused by MG treatment. The glyoxalase system detoxifies most cellular MG, and Glo-1 converts MG into a nontoxic hemithioacetal metabolite using glutathione (28, 29). Upregulation of Glo-1 reduced hyperglycemia-induced carbonyl stress, AGE accumulation, and oxidative stress in diabetic rats (35). Similar to previous studies (26–29), Glo-1 expression was markedly suppressed in NIT-1 cells following MG treatment, which promoted intracellular accumulation of MG. The results showed that Glo-1 silencing increased the intracellular concentration of MG and further reduced METTL3 expression and m^6^A RNA levels. Taken together, our findings suggest that the decreases in METTL3 expression and m^6^A content in NIT-1 cells after MG exposure may be attributed, at least in part, to the increase in intracellular MG accumulation.

In a rat model of MG-induced T2DM, MafA expression was reduced in pancreatic tissue (8, 36). We also confirmed that MafA mRNA and protein expression were decreased in MG-treated NIT-1 cells. In the present study, METTL3 silencing markedly decreased the half-life of MafA mRNA and protein levels in NIT-1 cells, indicating that MafA might be a direct target of METTL3. Moreover, upregulation of METTL3 reversed the MG-induced reduction in MafA expression. The results of the MeRIP-qPCR assay suggested that m^6^A levels in MafA mRNA were increased by METTL3 overexpression in MG-treated NIT-1 cells. MafA mRNA decay in NIT-1 cells induced by MG exposure was significantly ameliorated by upregulation of METTL3. Therefore, we conclude that MafA is a critical transcription factor regulated by METTL3 during MG-induced pancreatic β-cell damage.

MafA is not only a key activator of insulin transcription but also a master regulator of genes involved in maintaining β-cell function (37). Knockdown of MafA with siRNA led to impaired insulin secretion in EndoC-βH1 cells (a human-derived β-cell line) and human islets (38). Matsuoka et al. generated transgenic db/db mice that specifically overexpress MafA in islet β cells and found that these mice had significantly lower plasma glucose levels, higher plasma insulin levels, and augmented islet β-cell mass (39). This is consistent with our observations that MafA overexpression reversed the β-cell GSIS impairment caused by METTL3 silencing. MafA silencing significantly abolished the protective effects of METTL3 upregulation against GSIS reduction in MG-treated NIT-1 cells. Taken together, these data indicated that changes in MafA expression are associated with METTL3-regulated GSIS in NIT-1 cells.

Our study provides new insights into the cause of islet β-cell dysfunction in patients with diabetes. The decrease in pancreatic β-cell m^6^A levels could be partly attributed to MG accumulation in pancreatic islets during DM development. Increasing METTL3 expression in islet β cells may be a novel method for ameliorating MG-induced diabetic β-cell dysfunction. However, further animal and clinical studies are required to confirm this finding.

## Conclusion

In summary, the present study demonstrated, for the first time, the connection between METTL3-regulated m^6^A RNA levels and MG-induced pancreatic β-cell insulin secretion dysfunction. We found that treatment with MG reduced the m^6^A levels in β cells by decreasing METTL3 expression. Upregulation of METTL3 ameliorated MG-induced impairment of insulin secretion in pancreatic β cells by regulating MafA expression.

## Data Availability Statement

The raw data supporting the conclusions of this article will be made available by the authors, without undue reservation.

## Author Contributions

QH and S-JW designed the experiment and supervised the project. YC, X-MY, S-MZ, YS, and X-JM performed the experiments. X-MY, YW, and S-JW analyzed the data and drafted the manuscript. Y-JX, Y-JX, and QH were responsible for critical reading, editing, and revising the manuscript. All authors read and approved the final manuscript. All authors contributed to the article and approved the submitted version.

## Funding

This study was supported by grants from the Anhui Province Key Research and Development Projects (202004j07020050).

## Conflict of Interest

The authors declare that the research was conducted in the absence of any commercial or financial relationships that could be construed as a potential conflict of interest.

## Publisher’s Note

All claims expressed in this article are solely those of the authors and do not necessarily represent those of their affiliated organizations, or those of the publisher, the editors and the reviewers. Any product that may be evaluated in this article, or claim that may be made by its manufacturer, is not guaranteed or endorsed by the publisher.
