# Supplementary figures and images for: The m6A Methyltransferase METTL3 Ameliorates Methylglyoxal-Induced Impairment of Insulin Secretion in Pancreatic β Cells by Regulating MafA Expression
Source: Front Endocrinol (Lausanne). 2022 Jul 8;13:910868. doi: 10.3389/fendo.2022.910868 (PMC9304699; doi:10.3389/fendo.2022.910868)

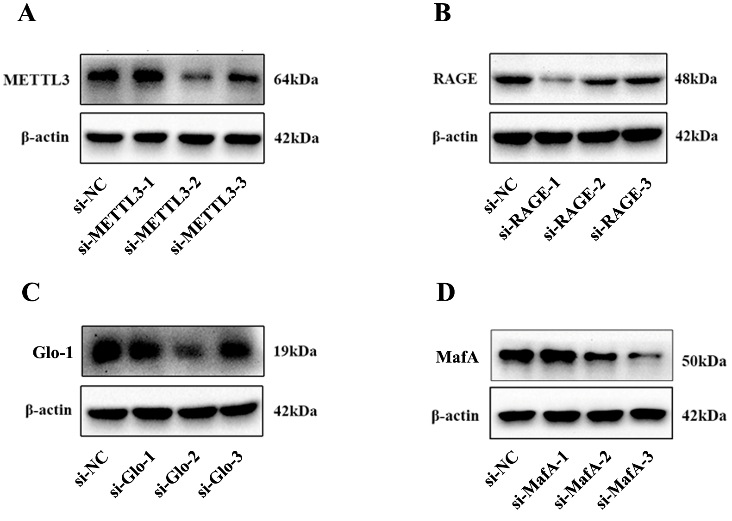


**Supplementary Figure 1** The knockdown efficiency of siRNAs METTL3, RAGE, Glo-1 and MafA.

Supplement: Supplementary file 1 [file DataSheet_1.docx]
